# The Clinical Significance of Drug–Food Interactions of Direct Oral Anticoagulants

**DOI:** 10.3390/ijms22168531

**Published:** 2021-08-08

**Authors:** Grzegorz Grześk, Daniel Rogowicz, Łukasz Wołowiec, Agnieszka Ratajczak, Wojciech Gilewski, Małgorzata Chudzińska, Anna Sinkiewicz, Joanna Banach

**Affiliations:** 1 Department of Cardiology and Clinical Pharmacology, Faculty of Health Sciences, Ludwik Rydygier Collegium Medicum in Bydgoszcz, Nicolaus Copernicus University in Toruń, Ujejskiego 75 Street, 85-168 Bydgoszcz, Poland; g.grzesk@cm.umk.pl (G.G.); lordtor111@gmail.com (Ł.W.); ratajczak.agnieszka06@gmail.com (A.R.); wgilewski@wp.pl (W.G.); joannna@op.pl (J.B.); 2 Department of Nutrition and Dietetics, Ludwik Rydygier Collegium Medicum in Bydgoszcz, Nicolaus Copernicus University in Toruń, Dębowa 3 Street, 85-626 Bydgoszcz, Poland; malgorzata.chudzinska@cm.umk.pl; 3 Department of Otolaryngology, Audiology and Phoniatrics, University Hospital No. 2, Collegium Medicum, Nicolaus Copernicus University in Toruń, Ujejskiego 75 Street, 85-168 Bydgoszcz, Poland; anna.sinkiewicz@wp.pl

**Keywords:** resveratrol supplementation, dabigatran, rivaroxaban, apixaban, edoxaban, betrixaban, dietary supplements, food interaction, treatment

## Abstract

Cardiovascular diseases are the most common cause of death in the world. For almost 60 years, vitamin K antagonists (VKAs) were the mainstay of anticoagulation therapy, but in recent years direct oral anticoagulants (DOACs) have become the anticoagulant treatment of choice. DOACs were initially considered drugs with no significant food interactions; however, clinical observations from daily practice have proved otherwise as interactions with food ingredients have been reported. Food, dietary supplements or herbs may contain substances that, when administered concomitantly with DOACs, can potentially affect the plasma concentration of the drugs. The aim of this paper was to evaluate the clinical significance of drug–food interactions of DOACs, such as dabigatran, rivaroxaban, apixaban, edoxaban and betrixaban. Patients treated with anticoagulants should avoid products containing *St. John’s wort* and take special care with other food ingredients. As the interest in dietary supplements is on the rise, healthcare providers can contribute to the development of well-designed clinical trials on interactions between DOACs and food, and distribute sufficient knowledge about the proper use of these supplements among patients.

## 1. Introduction

Each year, 18 million people die of cardiovascular diseases, which account for 31% of all global deaths. Cardiovascular diseases are the most common cause of death in the world [1]. Additionally, every year about 290,000 people die of atrial fibrillation (AF), which is the most common sustained cardiac arrhythmia worldwide [1,2]. AF affects 2–4% of adults and causes approximately 20% of all ischemic strokes [2]. It is estimated that in the future its incidence may increase threefold due to an aging population and comorbidities such as hypertension, diabetes, heart failure, coronary artery disease, chronic kidney disease, obesity and obstructive sleep apnea [2]. For almost 60 years, vitamin K antagonists (VKAs) were the mainstay of anticoagulation therapy [3]. In 2008, a new class of drugs was introduced in the markets of the European Union and the United States, which was a promising alternative to VKAs in the prevention of embolic complications in non-valvular AF, as well as in the treatment of patients with deep vein thrombosis and pulmonary embolism [4]. These were new-generation oral anticoagulants, originally referred to as new/novel oral anticoagulants (NOACs) and now as direct oral anticoagulants (DOACs) [5]. They act as direct factor Xa inhibitors (rivaroxaban, apixaban, edoxaban and betrixaban) or direct thrombin inhibitors (dabigatran) [2,6]. Their anticoagulant effect is more predictable and stable (i.e., less dependent on interactions with food, herbal supplements and other drugs) compared to warfarin and acenocoumarol [7]. The use of DOACs does not require individual dose adjustment or routine monitoring of blood coagulation parameters, such as the international normalized ratio (INR), activated partial thromboplastin time (APTT) and thrombin time [2]. Using VKA therapy, the therapeutic INR range of 2.0 – 3.0 is recommended in the prevention of embolic complications in non-valvular AF, in the treatment of deep vein thrombosis and pulmonary embolism. It is recommended that time in therapeutic range (TTR) be >70% during VKA therapy, which in the context of significant dietary interactions and individual pharmacokinetic profiles mandates frequent INR control. Therefore, thecost-effectiveness and safety of long-term VKA treatment are considerably lower [2]. However, contrary to common belief, some pharmacokinetic variations secondary to interactions with food, herbal supplements and other drugs should still be considered in patients treated with DOACs [8,9].

## 2. Bioavailability and Metabolism of DOACs

**Figure 1:**
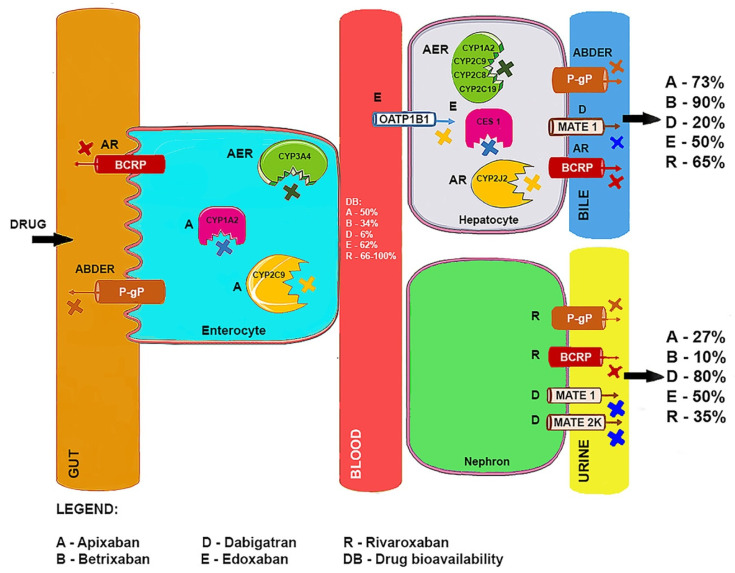
The absorption and metabolism of DOACs [10,14,15,16,17,18,19,20].

DOAC bioavailability is affected by the renal excretion of drugs, gastrointestinal and renal re-secretion by ABC transporters as well as drug metabolism by cytochrome P450 (CYP) enzymes [10]. P-glycoprotein (P-gp/ABCB1/multidrug resistance 1 (MDR1)) and breast cancer resistance protein (BCRP/ABCG2/ABCP) belong to the family of ABC transporters that protect cells from toxic effects of substances by removing them against the concentration gradient through the cell membrane, consuming energy from ATP hydrolysis [11]. P-gp and BCRP are present on the apical membrane of cells in several normal human organs (liver, kidneys, adrenal gland) and tissue junctions (blood–brain barrier, intestine, placenta, blood–testis and blood–ovarian barriers) [11]. They protect the human organism against the detrimental effects of xenobiotics, but by the same token, they take part in drug–drug and drug–food interactions [12]. In addition, food and drugs affect the activity of cytochrome P450 enzymes involved in drug metabolism, also contributing to important interactions [13]. In fact, DOACs, except for dabigatran and betrixaban, are mainly metabolized by the CYP3A4 isoform, which is present both in the gut and the liver [14]. Concomitant use of DOACs with other drugs, certain food or herbs may affect the activity of drug transporters and metabolizing enzymes, which may result in pharmacokinetic interactions leading to low efficacy or unexpected toxicities [14]. The absorption and metabolism of DOACs are presented in Figure 1.

Abbreviations: BCRP, breast cancer resistance protein; MATE1, multidrug and toxin extrusion protein 1; MATE2K, multidrug and toxin extrusion protein 2K; P-gp, P-glycoprotein; CYP1A2, cytochrome P450 1A2; CYP3A4, cytochrome P450 3A4; CYP2C8, cytochrome P450 2C8; CYP2C9, cytochrome P450 2C9; CYP2C19, cytochrome P450 2C19; CYP2J2, cytochrome P450 2J2; CES1, carboxylesterase 1. 

Dabigatran etexilate is a prodrug, which is hydrolyzed to dabigatran after oral administration [10]. It achieves peak plasma level approximately 2 h after ingestion and its absolute oral bioavailability is 6.0% [17]. The bioavailability of dabigatran increases by as much as 75% when the granules are taken without a protective capsule compared to the intact capsule. Therefore, the drug should not be crushed or chewed [21]. Due to the fact that dabigatran capsules are designed for release in the stomach and absorption in the proximal small intestine, it must not be administered in patients receiving nutrition and oral medications via nasogastric, gastrostomy or jejunostomy tubes [22]. Concomitant food ingestion does not affect dabigatran bioavailability, but delays its maximum plasma concentration (Cmax) by 2 h [21]. Dabigatran is not metabolized by cytochrome P450 enzymes [23]. It is mainly eliminated by the kidneys and is the only DOAC that can be removed by hemodialysis [21]. The elimination of dabigatran (80% of dabigatran etexilate oral dose) is performed primarily via the kidneys probably by multidrug and toxin extrusion protein 1 (MATE1) and multidrug and toxin extrusion protein 2K (MATE2K), which could play an important role in its renal clearance and drug–drug or drug–food interactions. Dabigatran is a relatively poor P-gp substrate in the kidneys [18]. However, dabigatran etexilate (prodrug) could be subject to changes in absorption as it is a substrate for P-gp, which plays a key role in regulating its intake in the intestinal wall. Hence, P-gp inhibitors can increase and P-gp inducers can reduce its absorption, respectively [23].

Rivaroxaban is an active drug whose bioavailability is 80–100% and peak plasma level is reached after 2–4 h [24]. However, on an empty stomach its bioavailability is 66%. When taken as a 20 mg tablet after a meal, an increase in mean area under the curve (AUC) of 39% was observed compared to ingestion on an empty stomach, indicating almost complete absorption and high oral bioavailability [24]. With a 10 mg dose, no significant effect of food on the pharmacokinetics was observed [25]. The bioavailability of rivaroxaban is also dependent on the site of drug release in the gastrointestinal tract; thus, the administration of rivaroxaban distal to the stomach is not recommended. The release of rivaroxaban in the proximal small intestine causes a decrease in AUC and Cmax by 29% and 56%, respectively. Drug release in the distal small intestine, or ascending colon, leads to a further reduction in its exposure [26]. For rivaroxaban, there is no contraindication to crushing tablets and mixing them with water or apple mousse to facilitate drug delivery [27]. Approximately 30% of rivaroxaban is excreted unchanged by the kidneys with P-gp and BCRP, and 6% via glomerular filtration. Thirty-two percent is metabolized in the liver by CYP3A4, CYP3A5 and CYP2J2 and 14% via non-CYP-mediated hydrolysis of the amide bonds [16,19]. The interaction of rivaroxaban with inhibitors/inducers of both CYP enzymes and the transport proteins P-gp or BCRP (or both) may impair the effective bioavailability of the drug, and therefore cause a clinically relevant potential drug–drug or drug–food interaction [16,24].

Apixaban has an oral bioavailability of about 50% and is absorbed primarily in the upper gastrointestinal tract (duodenum, jejunum and ileum), with decreased absorption at more distal sites [15]. The drug reaches its peak concentration 1 to 4 h after ingestion [15]. Apixaban can be taken with or without food; however, after food ingestion Cmax is reduced by 15% and time to maximum plasma concentration (Tmax) is shortened [28]. Alternative methods of drug administration in patients fed via a nasogastric feeding tube and with swallowing problems have also been tested. When 10 mg of apixaban (2 × 5 mg tablets) was crushed and mixed with 30 mL of water, Cmax and AUC met the criteria for bioequivalence compared to the administration of whole tablets. When apixaban was crushed and mixed with 30 g of applesauce, Cmax and AUC decreased by 21.1% and 16.4%, respectively [28]. However, this reduction was not considered clinically relevant [29]. The drug is actively taken up by P-gp and BCRP located in the intestine and bile ducts [30]. Apixaban is mainly eliminated via excretion into the intestinal tract, and 27% of the drug is excreted unchanged in urine [31]. Less than 32% is metabolized by cytochrome P450 enzymes, mainly by CYP3A4, but also by CYP1A2, CYC2J2, CYC2C8, CYC2C9 and CYC2C19 [32,33]. Its concomitant use with inducers/inhibitors of P-gp, BCRP or CYP3A4 may have an effect on apixaban plasma concentration [29,34].

Edoxaban reaches peak plasma level 1–2 h after ingestion, and its bioavailability is 62%. It is predominantly absorbed from the upper gastrointestinal tract and food does not affect its absorption [35]. Edoxaban tablets can be crushed and administered either in apple puree and taken orally or as a water suspension via a nasogastric tube [36]. The absorption is mediated by P-gp. Approximately 50% of edoxaban is eliminated by the kidneys and the remaining through the liver and biliary excretion. The drug is mainly excreted unchanged in urine and bile, and its hepatic metabolism accounts for 10%. Edoxaban undergoes limited metabolism mediated by carboxylesterase 1 (CES1), CYP3A4 and CYP3A5, enzymatic hydrolysis and glucuronidation. Hepatic impairment (Child–Pugh class A and B) does not significantly change the peak plasma concentration or total edoxaban exposure and active metabolites are unlikely to contribute significantly to anticoagulant activity [35]. Inducers or inhibitors of both P-gp and metabolic enzymes could change edoxaban plasma concentration and therefore could potentially cause a clinically meaningful effect [35,37,38,39,40].

Betrixaban plasma concentration peaks after 3–4 h, and the terminal plasma half-life of the drug is 37 h. The bioavailability of betrixaban administered orally accounts for 34% and is affected by fatty foods, which reduce both Cmax and AUC by 50%. Betrixaban is not metabolized by cytochromes. Ninety percent of the drug is excreted unchanged in bile via P-gp efflux pump. Betrixaban has the lowest renal clearance of all DOACs [41]. Its concomitant use with inducers or inhibitors of P-gp may affect the plasma concentration of the drug [41,42,43,44].

## 3. Use of DOACs with Proton Pump Inhibitors and Activated Charcoal

Gastrointestinal bleeding is a common side effect associated with anticoagulant therapy. Apixaban and dabigatran are associated with a lower overall bleeding risk compared to warfarin or rivaroxaban. Apixaban carries a lower risk of severe gastrointestinal bleeding than dabigatran [45]. In a 2018 study on DOACs, the authors emphasize that long-term therapy may require more effective stomach protection through the use of proton pump inhibitors (PPIs) [46]. PPIs have also been shown to be useful in alleviating indigestion associated with dabigatran [47]. Nonetheless, there have been several reports of adverse interactions between PPIs and anticoagulants associated with decreased anticoagulant activity. This interaction has been demonstrated in patients treated with dabigatran, which requires an acidic absorption environment and is therefore conserved with tartaric acid [46,48]. Concomitant administration of antacid has led to an approximately 20% reduction indabigatran absorption, but this is not considered to be of clinical relevance. The pharmacokinetics of rivaroxaban, apixaban and edoxaban are not affected by drugs that increase the gastric pH [48].

In the event of DOAC overdose, the use of activated charcoal may be considered. In vitro data indicate that dabigatran can be effectively absorbed by activated carbon. Importantly, the administration of activated charcoal is recommended in the event of bleeding if no more than 2 h have passed since the last dose [49]. A study conducted among healthy volunteers also showed that the administration of activated charcoal up to 6 h after a single dose of 20 mg apixaban reduced its exposure to apixaban and facilitated the elimination of this drug [50]. It has been shown that the administration of activated charcoal within 8 h of rivaroxaban intake significantly decreases DOAC plasma concentration [51].

## 4. DOAC Treatment in Patients after Gastrointestinal Surgery

Patients after gastrointestinal surgery treated with DOACs should be monitored more carefully. The therapy could be continued provided that the measured peak plasma and trough concentrations correspond to values expected in the general population [48]. It is recommended to avoid rivaroxaban treatment in patients undergoing gastrectomy, yet patients who have sustained major distal bowel resection could be treated with this agent. Dabigatran should be avoided in patients who underwent gastrectomy or major proximal or distal intestinal resections, according to isolated reports [22]. There is uncertainty about the efficacy of apixaban in patients after gastrointestinal operation [52,53,54]. However, apixaban could be a therapeutic option for patients who have undergone gastric procedures (sleeve gastrectomy, laparoscopic adjustable gastric banding and laparoscopic Roux-en-Y gastric bypass surgery) based on the only available study on the pharmacology of apixaban after bariatric surgery and theoretical knowledge that apixaban absorption is pH independent [22,54]. Anticoagulation efficacy data for edoxaban and betrixaban in the setting of gastrointestinal surgeries are not available.

## 5. Diet with DOACs

Some authors suggest that intermittent fasting can be a method of prevention of cardiovascular diseases [55]. This suggestion is based on a slight decrease in LDLcholesterol concentration observed after fasting, but so far it has not been confirmed in direct clinical trials. Moreover, from the point of view of pharmacokinetics, prolonged fasting in patients on active pharmacological treatment may be dangerous due to the possible changes in drug absorption leading to ineffective therapy and therefore potentially to myocardial infarction, stroke and other thrombotic events. For a rivaroxaban dose of 15 mg or more, the relation between food intake and drug absorption is significant; thus, this drug has to be taken with food only, but there are no differences in the absorption of lower doses [24]. However, fasting or irregular eating may cause dyspeptic symptoms [56]. Dabigatran capsules contain a tartaric acid core, which is responsible for 5–10% incidence of dyspepsia [57,58]. Administering dabigatran while fasting may increase the adverse effect of the drug on the digestive system, which may lead to treatment discontinuation.In patients receiving DOACs, a regular meal schedule and diet composition consistent with the guidelines on cardiovascular disease prevention should be recommended.

Studies on interactions between apixaban, edoxaban, dabigatran and individual macronutrients showed that the presence of proteins, fats and carbohydrates did not significantly affect the bioavailability of these drugs [21,28,35]. However, the results of an in vitro study by Raiola et al. showed that the presence of insoluble and soluble fiber as well as cellulose may cause a decrease in the bioavailability of dabigatran, rivaroxaban and apixaban. The presence of a high amount of insoluble and soluble dietary fiber significantly decreased DOAC bioavailability. However, a low or moderate amount of fiber did not have a significant effect on the bioavailability of DOACs, i.e., when they were a component of a balanced meal containing all the macronutrients. The study results suggest that it may be necessary to maintain a time interval between taking DOACs and a meal containing a high amount of cellulose and inulin. The authors of the study emphasize that further in vivo research is needed to evaluate the effect of dietary fiber on the bioavailability of anticoagulants [59].

## 6. DOAC Interaction with Dietary Supplements

In order to reduce the risk of cardiovascular diseases, the European Society of Cardiology and American Heart Association recommend the following:

— The right daily intake of calories should be based on weight, age and physical activity level;
— Choosing fiber-rich whole grains for most grain servings, nuts and legumes;
— Eating a variety of vegetables, fruits, skinless poultry and fish and preparing them in healthy ways;
— Limiting saturated fat, trans fat, sodium, red meat, sweets and sugar-sweetened beverages;
— Drinking alcohol in moderate amounts and avoiding smoking [60,61,62].

The United States (US) Food and Drug Administration (FDA) defines dietary supplements as oral products that contain ingredients including vitamins, minerals, amino acids, herbs or botanicals, as well as other substances that can be used to supplement the diet [63]. On the other hand, the European law (Directive2002/46/CE) defines them as vitamins, minerals, herbs and other natural products marketed in dosage forms such as tablets, pills or ampoules of liquids. Due to the lack of awareness and knowledge, patients misinterpret the recommendations, keeping a diet or using dietary supplements that may cause relevant clinical interactions with anticoagulants. Until recently, warfarin was the most commonly used anticoagulant drug [3]. Simultaneous consumption of foods rich in vitamin K (lettuce, broccoli, spinach, green peas) and herbs in the form of supplements or infusions (e.g., *St. John’s wort*, *Echinacea*) was associated with a risk of reduced therapeutic effect of VKAs [64]. Currently, DOACs are the most frequently prescribed anticoagulants [65,66]. In the US, 30% of patients treated with warfarin [67] and 20% of patients treated with apixaban (the most commonly used DOAC) take nutritional supplements on a regular basis [68]. Patients treated with apixaban most commonly use (daily or on most days): vitamin D, calcium, fish oil/omega-3 fatty acids/cod liver oil, B vitamins, vitamin E and various herbal compounds. Respondents also reported consuming herbal teas (11.1%), turmeric (9%), *St John’s wort* (<1%) and other herbal products (Chinese herbs, ginger, ginkgo biloba—all fewer than 5%), which potentially modify apixaban exposure [68]. Worldwide sales of supplements are steadily increasing, and sales of herbal medicines in the US have doubled in the last 20 years [69]. When choosing these products, patients often follow the opinion of pharmacists, who do not always have sufficient knowledge about their indications [69]. Dietary supplements are used in the treatment of cardiovascular diseases such as hypertension, hyperlipidemia, coronary artery disease, stroke and peripheral arterial disease. In addition, they delay the aging process and reduce the risk of dementia [70,71,72]. However, their use alongside DOAC therapy carries the risk of bleeding or a reduction in the therapeutic effect (Table 1). Some of these agents have antiplatelet effects, which in conjunction with DOACs can potentially significantly increase the risk of bleeding, as is obviously the case when combining anti-platelet drugs with DOACs [73].

The administration of all available DOACs and *St. John’s wort* (a strong inducer of CYP3A4 and P-gp) may reduce DOAC plasma concentration, which may in turn decrease the anticoagulant effect [10,26,29,42,85,86,87]. Nevertheless, in the available literature, there are no studies of interactions in humans between DOACs and food ingredients that are inhibitors of P-gp, BCRP or CYP3A4. Thus far, Maadarani et al. have described the case of an 80-year-old man with normal renal function and a 4-year history of chronic non-valvularAF, who was treated with dabigatran at a dose of 110 mg. The patient had fatal bleeding after taking a boiled mixture of ginger and cinnamon twice daily for 3 days before admission to hospital [88]. As shown in studies of interactions between DOACs and other drugs in humans, concomitant administration of strong inhibitors/inducers of P-gp, BCRP or CYP3A4 with DOACs may cause significant interactions [14]. The concomitant use of P-gp inhibitors with DOACs may increase the plasma levels of the drugs. CYP3A4 inhibitors could increase plasma concentrations of edoxaban, rivaroxaban and apixaban. Moreover, the use of P-gp and BCRP inhibitors or CYP3A4 may increase plasma concentrations of apixaban and rivaroxaban [14]. In humans, certain food ingredients or medicinal herbs may potentially affect P-gp, BCRP and cytochrome P450 enzymes, and influence DOAC levels (Table 1). A decrease in DOAC concentration may increase the risk of thromboembolic events, while an increase in DOAC concentration may increase the risk of bleeding [89].

## 7. Clinical Implications

Dietary supplements or herbs may contain several substances that have the potential to affect DOAC levels [20]. Furthermore, some patients take more than one dietary supplement or herb [90]. This creates the risk of additive or hyper-additive synergism due to their strong influence on at least two targets involved in the absorption or metabolism of DOACs (ABC transporters and cytochrome P450 isoforms) [10,91]. DOACs have a relatively narrow therapeutic window, which may contribute to the fact that substances with a weak or moderate effect on ABC transporters or cytochrome P450 enzymes may still adversely affect their pharmacokinetics and cause clinically significant interactions. Additionally, several different dietary products or herbs may be a source of a given compound, which, when administered concomitantly, increases the effective dose of the substance taken and its bioavailability. Studies evaluating the effect of different substances on ABC transporters and cytochrome P450 isoforms have been partially conducted in animals, in which the expression of the ABC transporters and cytochrome P450 isoforms differs from that in humans. In addition, gene polymorphism is also observed in humans [11,12,92]. Therefore, there is a need for human studies to evaluate their safety at recommended doses with DOACs.

On one hand, apixaban demonstrates the most favorable profile of fluctuations in plasma concentration by the geometric coefficient of variation among all DOACs [7]. On the other hand, apixaban is similar to warfarin in cytochrome P450 metabolism, which potentially increases the possibility of interactions between this drug and dietary components [14]. In the case of dabigatran and betrixaban, the risk of interactions can theoretically be reduced by a time interval between the administration of these DOACs and the intake of foods that affect P-gp, assuming that P-gp blockers are reversible and short-lived. The time interval should be at least 4 h due to the time required to reach the peak plasma level. Nevertheless, patients treated with DOACs should limit their consumption of dietary supplements and avoid multi-ingredient food products. The concomitant consumption of DOACs with these substances may require periodic measurements of DOAC plasma levels.

## 8. Compliance

Physicians’ clinical recommendations, which are based on the knowledge of drug indications, pharmacokinetics and pharmacodynamics, form a foundation of a safe and effective therapy. Unfortunately, patients’ compliance with these recommendations is sometimes a serious problem. If patients do not take medications as recommended, the expected therapeutic effect may not be achieved. The assessment conducted by the WHO in developed countries shows that only about 50% of patients with chronic diseases follow recommendations. The term “compliance” usually implies the passive participation of the patient, which consists only of adapting to the doctor’s instructions, which, in long-term treatment, often fails. Patients must also be an active party in this process, and the treatment strategy should result from an agreement with their doctors (concordance). The preferred term is “adherence to a therapeutic plan” (adherence). The more patients understand the meaning of diagnostic and therapeutic measures, the more they accept these measures, which significantly improves the effectiveness of therapy [93].

Non-compliance in patients with AF treated with DOACs is due to, among others, the misunderstanding of medical prescriptions, fear of side effects, economic considerations, or the lack of conviction that therapy is actually necessary [94]. In large populations, objective verification (e.g., testing the concentration of a drug or its metabolite) is practically impossible due to costs, logistic difficulties and a lack of resources. Nevertheless, the assessment of medication intake according to the therapeutic plan, based on the patient’s interview, may have limited credibility, as some subjects may provide information expected by doctors, but not necessarily true, and some patients refuse to answer at all [95].

When DOACs are used, greater compliance can be expected during treatment with rivaroxaban, edoxaban or betrixaban, which is taken once a day, as opposed to dabigatran and apixaban, which require administration every 12 h [94]. However, apixaban demonstrates the most optimal variation in plasma concentration by the geometric coefficient of variation compared with dabigatran and rivaroxaban [7].

## 9. Summary

Oral direct anticoagulants do not require routine INR control, and there are fewer interactions with food than with vitamin K antagonists. However, this does not mean that DOACs are free of drug–food interactions. Patients treated with anticoagulants should avoid products containing *St. John’s wort* and take special care with other food ingredients. As the use of dietary supplements is extensive and growing, healthcare providers can contribute to the development of well-designed clinical trials on interactions between DOACs and food, as well as distribute sufficient knowledge about the proper use of these supplements among patients. Further research is also needed to evaluate the effect of dietary fiber on the bioavailability of anticoagulants. High-fat foods are known to reduce the overall concentration of betrixaban. In contrast, rivaroxaban 20 mg is better absorbed and has almost 100% bioavailability when taken with food. Patients with dyspepsia can be treated with apixaban, rivaroxaban and edoxaban because there are no contraindications for the pills to be crushed and mixed with water or apple mousse. DOAC therapy in patients after gastrointestinal tract resection should be adjusted depending on the type and extent of surgery. Unfortunately, oral direct anticoagulants are not free of side effects, which include gastrointestinal and intracranial hemorrhage. Long-term DOAC therapy may require gastric protection through the use of PPIs. Patients’ adherence to therapeutic recommendations is a substantial problem. If the patient does not regularly take medication, the expected therapeutic effect may not be achieved. When DOACs are used, greater adherence can be expected during treatment with rivaroxaban, edoxaban or betrixaban, which is taken once a day, as opposed to dabigatran and apixaban, which require administration every 12 h. Apixaban demonstrates the most favorable fluctuations in plasma concentration by the geometric coefficient of variation among all DOACs.

## Figures and Tables

**Table 1:** Potential effect of food ingredients on DOACs [10,20,74,75,76,77,78,79,80,81,82,83,84].

Substance	Source of Substance	Mechanism of Action	Effect on DOACs
Alpha-lipoic acid		Exhibits antiplatelet activity	Potentially increases the risk of bleeding when used concomitantly with DOACs
Apigenin *	*M. chamomilla* (*Camomile*) *M. officinalis* (*Lemon balm*) *P. emblica* (*Emblic myrobalan*) *S. costus* (*Costus*)	Inhibition of cytochrome P450 (1A2, 2C9, 2C19, 3A4), P-gp and BCRP Exhibits antiplatelet activity	Potentially increases plasma concentration of all DOACs Potentially increases the risk of bleeding when used concomitantly with DOACs
α-Asarone	*A. calamus* (*Sweet flag*) *A. gramineus* (*Japanese sweet flag*)	Inhibition of cytochrome P450 (1A1, 3A4, 2B6, 2C8, 2C9, 2C19, 2D6, 2E1) and P-gp	Potentially increases plasma concentration of all DOACs
β-Asarone	*A. calamus* (*Sweet flag*) *A. gramineus* (*Japanese sweet flag*) *R. acori*	Inhibition of CYP3A4 and P-gp	Potentially increases plasma concentration of all DOACs
Avenanthramide (A, B, C) *	*A. sativa* (*Oat*)	Inhibition of P-gp	Potentially increases plasma concentration of all DOACs
Bacoside (A, B) *	*B. monnieri*(*Water hyssop*)	Inhibition of cytochrome P450 (1A2, 3A4, 2C9, 2C19)	Potentially increases plasma concentration of rivaroxaban, apixaban and edoxaban
Berberine *	*C. chinensis* (*Chinese goldthread*) *C. japonica* (*Camellia*)	Inhibition of cytochrome P450 (1A2, 3A4, 2C9, 2D6), P-gp and BCRP	Potentially increases plasma concentration of all DOACs
Bilobalide *	*G. biloba* (*Ginko*)	Inhibition of cytochrome P450 (1A1, 1A2, 3A4, 2B6, 2C9, 2E1) and P-gp	Potentially increases plasma concentration of all DOACs
Biochanin A *	*T. pratense* (*Red clover*)	Inhibition of CYP3A4, P-gp and BCRP Exhibits antiplatelet activity. May enhance effects of anticoagulant	Potentially increases plasma concentration of all DOACs Potentially increases the risk of bleeding when used concomitantly with DOACs
Caffein *	*C. arabica* (*Arabian coffee*) *I. paraguariensis* (*Yerba mate*) *P. cupana* (*Guaraná*) *T. cacao* (*Cacao tree*) *C. sinensis* (*Chinese liver fluke*)	Inhibition of cytochrome P450 (1A2, 3A4) and BCRP	Potentially increases plasma concentration of rivaroxaban, apixaban and edoxaban
Capsaicin *	*Capsicum* (*Chili peppers*)	Induction of CYP3A4 and inhibition of P-gp	Potentially increases plasma concentration of dabigatran and betrixaban
Carbolines (Harmine) *	*L. meyenii* (*Maca*) *M. pruriens* (*Velvet bean*) *P. harmala* (*Wild rue*)	Inhibition of cytochrome P450 (1A1,1A2, 2C9, 2C19, 2D6, 2E1) and BCRP	Potentially increases plasma concentration of rivaroxaban, apixaban and edoxaban
Casticin *	*V. agnus-castus* (*Chaste tree*)	Inhibition of cytochrome P450 (3A4, 2C9)	Potentially increases plasma concentration of rivaroxaban, apixaban and edoxaban
Catechin *	*C. rotundus* (*Coco-grass*) *L. bicolor* (*Shrub lespedeza*) *M. chamomilla* (*Camomile*) *T. cacao* (*Cacao tree*)	Inhibition of cytochrome P450 (1A2, 3A4, 2C9) and P-gp	Potentially increases plasma concentration of all DOACs
Chebulagic acid *	*T. chebula* (*Chebulic myrobalan*) *P. emblica* (*Emblic myrobalan*)	Inhibition of P-gp	Potentially increases plasma concentration of all DOACs
Chicoric acid, Alkylamides *	*G. Echinacea* (*nine* *known species*)	Inhibition of CYP3A4	Potentially increases plasma concentration of rivaroxaban, apixaban and edoxaban
Cinnamaldehyde *	*C. wilsonii*	Inhibition of cytochrome P450 (1A2, 2E1) and P-gp	Potentially increases plasma concentration of all DOACs
Coniferyl ferulate *	*A. sinensis*(*Dong quai*)	Inhibition of cytochrome P450 (3A4, 2D6) and P-gp	Potentially increases plasma concentration of all DOACs
Coraria lactone	*Alismaorientalis* (*Alismataceae*)	Induction of P-gp	Potentially decreases plasma concentration of all DOACs
Coumarin *	*A. hippocastanum* (*Horse chestnut*) *Cassia cinnamon* (*Cinnamon*)	Exhibits antiplatelet activity	Potentially increases the risk of bleeding when used concomitantly with DOACs
Crocin *	*C. sativus* (*Saffron*)	Inhibition of cytochrome P450 (3A4, 3A5, 3A7,2B6) and P-gp	Potentially increases plasma concentration of all DOACs
Curcumin *	*C. longa* (*Turmeric*)	Inhibition of cytochrome P450 (1A2, 3A4, 2B6, 2C9, 2D6) and P-gp, induction/inhibition of BCRP Exhibits antiplatelet activity. May enhance effects of anticoagulant.	Potentially increases plasma concentration of all DOACs Potentially increases the risk of bleeding when used concomitantly with DOACs
Decursin *	*A. gigas* (*Korean angelica*)	Inhibition of cytochrome P450 (1A1, 1A2) and P-gp	Potentially increases plasma concentration of all DOACs
Dehydroepiandrosterone *	*Soybean* (*Glycine max*)	Inhibition of CYP3A4	Potentially increases plasma concentration of rivaroxaban, apixaban and edoxaban
Delphinidin *	*V. uliginosum L.* (*Bog bilberry*)	Inhibition of cytochrome P450 (3A4, 2B6, 2C9), and BCRP	Potentially increases plasma concentration of rivaroxaban, apixaban and edoxaban
Ellagic acid *	*T. chebula* (*Chebulic myrobalan*) *P. emblica* (*Emblic myrobalan*)	Inhibition of BCRP	Potentially increases plasma concentration of rivaroxaban and apixaban
Ent-kaurane *	*C. tonkinensis*	Inhibition of P-gp	Potentially increases plasma concentration of all DOACs
Ephedrine *	*Angelica sinensis*(*Apiaceae*)	Inhibition of P-gp	Potentially increases plasma concentration of all DOACs
Epicatechin gallate (ECG) * Epigallocatechin-3-gallate (EGCG) *	*C. sinensis* (*Chinese liver fluke*)	Inhibition of cytochrome P450 (1A1, 1A2, 3A4) and P-gp	Potentially increases plasma concentration of all DOACs
Eucalyptus oil	*E. globulus* (*Eucalyptus*)	Inhibition of cytochrome P450 (1A2, 2C9, 2C19, 3A4)	Potentially increases plasma concentration of rivaroxaban, apixaban and edoxaban
Feverfew oil	T. parthenium *(*Feverfew*)	Inhibition of cytochrome P450 (1A2, 2C9, 2C19, 3A4) Exhibits antiplatelet activity	Potentially increases plasma concentration of rivaroxaban, apixaban and edoxaban Potentially increases the risk of bleeding when used concomitantly with DOACs
Galantamine *	*G. nivalis* (*Snowdrop*) *G. woronowii* (*Green snowdrop*) *L. radiata* (*Red spider lily*) *N. confusus* (*Lily of Mary*) *P. illyricum*	Inhibition of P-gp	Potentially increases plasma concentration of all DOACs
Gallic acid *	*M. pruriens* (*Velvet bean*) *P. emblica* (*Emblic myrobalan*) *T. chebula* (*Chebulic myrobalan*)	Inhibition of CYP3A4 and P-gp	Potentially increases plasma concentration of all DOACs
Gingerol *	*A. melegueta* (*Melegueta pepper*) *Z. officinaleRosc* (*Ginger*)	Inhibition of cytochrome P450 (3A4, 2C9, 2C19) and P-gp Exhibits antiplatelet activity	Potentially increases plasma concentration of all DOACs Potentially increases the risk of bleeding when used concomitantly with DOACs
Ginkgolide A, B *	*G. biloba* (*Ginkgo*)	Inhibition of cytochrome P450 (3A4, 2C9) and induction of P-gp Exhibits antiplatelet activity	Potentially decreases plasma concentration of dabigatran and betrixaban Potentially increases the risk of bleeding when used concomitantly with DOACs
Ginsenoside Rb, Rd *	*P. ginseng* (*Ginseng*)	Inhibition of cytochrome P450 (3A4, 2C9), BCRP Exhibits antiplatelet activity	Potentially increases plasma concentration of rivaroxaban, apixaban and edoxaban Potentially increases the risk of bleeding when used concomitantly with DOACs
Glabridin *	*G. glabra* (*Licorice*)	Inhibition of CYP3A4 and P-gp	Potentially increases plasma concentration of all DOACs
Grapefruit juice	*C. paradisi* (*Grapefruit*)	Inhibition of CYP3A4 and P-gp	Potentially increases plasma concentration of all DOACs
Guggulsterone *	*Guggul* (*Commiphoramukul*)	Induction CYP3A4 and inhibition of P-gp	Potentially increases plasma concentration of dabigatran and betrixaban
Honokiol *	*P. kaempferi* (*Pinaceae*)	Inhibition of P-gp	Potentially increases plasma concentration of all DOACs
Hydrastine *	*Hydrastis canadensis* (*Goldenseal*)	Inhibition of CYP3A4	Potentially increases plasma concentration of rivaroxaban, apixaban and edoxaban
Hyperforin, hypericin *	*H. perforatum* (*St. John’s wort*)	Induction of cytochrome P450 (1A2, 2C9, 3A4) and P-gp	Decreases plasma concentration of all DOACs Concomitant use with dabigatran and rivaroxaban should be avoided. Concomitant administration with edoxaban and apixaban should be used with caution (according to EHRA)
1,2,3,4,6-Penta-*O*-galloyl- -d-glucose *	*T. chebula* (*Chebulic myrobalan*)	Inhibition of P-gp	Potentially increases plasma concentration of all DOACs
E-Harpagoside *	*S. buergeriana* (*Buerger’s Figwort*)	Inhibition of P-gp	Potentially increases plasma concentration of all DOACs
Kavalactones *	*Piper methysticum* (*Kava*)	Inhibition of cytochrome P450 (1A2, 2C9, 3A4) and induction of P-gp	Potentially decreases plasma concentration of dabigatran and betrixaban
Lime extract	*C.aurantifolia* (*Lime*)	Inhibition of CYP3A4	Potentially increases plasma concentration of rivaroxaban, apixaban and edoxaban
Luteolin *	*L. bicolor* (*Shrub raspedeza*) *M. chamomilla* (*Camomile*) *M. officinalis* (*Lemon balm*) *P. emblica* (*Emblic myrobalan*) *R. officinalis* (*Rosemary*)	Inhibition of cytochrome P450 (1A2, 3A4, 2B6, 2C8, 2C9, 2C19, 2D6, 2E1) and P-gp Exhibits antiplatelet activity	Potentially increases plasma concentration of all DOACs Potentially increases the risk of bleeding when used concomitantly with DOACs
Malvidin 3-galactoside *	*V. angustifolium* (*Wild lowbush blueberry*)	Inhibition of cytochrome P450 (3A4, 2C9), BCRP, P-gp	Potentially increases plasma concentration of all DOACs
Malvidin 3-glucoside *	*V. angustifolium* (*Wild lowbush blueberry*) *V. uliginosum L.* (*Bog bilberry*)	Inhibition of cytochrome P450 (3A4, 2C9), BCRP, P-gp	Potentially increases plasma concentration of all DOACs
Mangiferin *	*M. indica* (*Mango*)	Inhibition of cytochrome P450 (1A1,1A2, 3A4, 2B6, 2C8, 2D6) and P-gp	Potentially increases plasma concentration of all DOACs
Myricetin *	*M. peregrina* (*Ben tree*) *R. nigrum* (*Blackcurrant*)	Inhibition of cytochrome P450 (1A2, 3A4, 2D6), BCRP and P-gp	Potentially increases plasma concentration of all DOACs
Naringenin	*L. bicolor* (*Shrub lespedeza*) *M. lucida* (*Brimstone tree*)	Inhibition of cytochrome P450 (3A4, 2C9, 2C19, 2E1), P-gp and BCRP	Potentially increases plasma concentration of all DOACs
Nobiletin *	*C. reticulata* (*Mandarin*)	Inhibition of CYP3A4, BCRP and P-gp	Potentially increases plasma concentration of all DOACs
Oleanolic acid *	*M. lucida* (*Brimstone tree*) *R. officinalis* (*Rosemary*)	Inhibition of cytochrome P450 (1A2, 3A4), BCRP and P-gp	Potentially increases plasma concentration of all DOACs
Omega-3 polyunsaturated fatty AIDS (n-3 PUFA)	*Fish oil*	Exhibits antiplatelet activity	Potentially increases the risk of bleeding when used concomitantly with DOACs
Paeoniflorin *	*Paeonia alba* (*Paeoniaceae*)	Induction of P-gp	Potentially decreases plasma concentration of all DOACs
p-Synephrine *	*C. aurantium* (*Bitter orange*)	Inhibition of CYP3A4 and P-gp	Potentially increases plasma concentration of all DOACs
Paeonol *	*P. lactiflora* (*Chinese peony*)	Inhibition of BCRP	Potentially increases plasma concentration of rivaroxaban and apixaban
Palmatine	*C. chinensis* (*Chinese goldthread*) *C. speciosa*	Induction of CYP3A4 and P-gp	Potentially decreases plasma concentration of all DOACs
Phellamurin	*Phellodendronwilsonii*(*Rutaceae*)	Inhibition of P-gp	Potentially increases plasma concentration of all DOACs
Phyllanthin *	*P. emblica* (*emblic myrobalan*)	Inhibition of CYP3A4 and P-gp	Potentially increases plasma concentration of all DOACs
Piperine *	*P. nigrum* (*Black pepper*) *P. longum* (*Long pepper*)	Inhibition of cytochrome P450 (3A4, 2C9, 2E1), P-gp and BCRP	Potentially increases plasma concentration of all DOACs
Polyphenols *	*Theaceae* (*Green tea leaf*)	Short-term inhibition and long-term induction of CYP3A4, induction of P-gp Exhibits antiplatelet activity	Potentially decreases plasma concentration of all DOACs Potentially increases the risk of bleeding when used concomitantly with DOACs
Prunus avium extract	*P. avium* (*Wild cherry*)	Inhibition of CYP3A4	Potentially increases plasma concentration of rivaroxaban, apixaban and edoxaban
Pyranocoumarins	*P. praeruptorum* (*Ningqianhu*)	Inhibition of P-gp	Potentially increases plasma concentration of all DOACs
Quercetin *	*A. melegueta* (*Melegueta pepper*) *C. sativus* (*Saffron*) *C. rotundus* (*Coco-grass*) *H. perforatum* (*St. John’s wort*) *I. paraguariensis* (*Yerba mate*) *L. meyenii* (*Maca*) *M. lucida* (*Brimstone tree*) *P. emblica* (*Emblic myrobalan*) *R. nigrum* (*Blackcurrant*) *S. costus* (*Costus*) *V. uliginosum L.* (*Bog bilberry*)	Inhibition of cytochrome P450 (1A1, 1A2, 3A4, 2C8, 2C9, 2C19, 2D6) and P-gp, induction of BCRP Exhibits antiplatelet activity	Potentially increases plasma concentration of dabigatran, edoxaban and betrixaban Potentially increases the risk of bleeding when used concomitantly with DOACs
Quercetin-3-*O*--Dglucuronide	*P. pterocarpum* (*Copperpod*)	Inhibition of CYP3A4 and P-gp	Potentially increases plasma concentration of all DOACs
Resveratrol *	*V. vinifera* (*Grape*)	Inhibition of cytochrome P450 (1A1, 1A2, 3A4, 2C8, 2C9, 2C19, 2D6) and P-gp, induction of BCRP	Potentially increases plasma concentration of dabigatran, edoxaban and betrixaban
Rosmarinic acid *	*M. officinalis* (*Lemon balm*) *M. spicata* (*Spearmint*) *R. officinalis* (*Rosemary*)	Inhibition of cytochrome P450 (3A4, 2C9, 2C19, 2D6, 2E1), P-gp and BCRP	Potentially increases plasma concentration of all DOACs
Rutin *	*H. perforatum* (*St. John’s wort*) *L. bicolor* (*Shrub laspedeza*) *M. chamomilla* (*Camomile*) *M. flexuosa* (*Moriche palm*) *M. lucida* (*Brimstone tree*) *M. peregrina* (*Ben tree*) *V. uliginosum L.* (*Bog bilberry*)	Inhibition of CYP3A4, P-gp and BCRP Exhibits antiplatelet activity	Potentially increases plasma concentration of all DOACs Potentially increases the risk of bleeding when used concomitantly with DOACs
Safranal *	*C. sativus* (*Saffron*)	Inhibition of P-gp and BCRP	Potentially increases plasma concentration of all DOACs
Salidroside *	*R. rosea* (*Golden root*)	Inhibition of CYP3A4 and P-gp	Potentially increases plasma concentration of all DOACs
S-allyl-l-cysteine sulphoxides (alliin) *	*A. sativum* (*Garlic*)	Induction of P-gp and BCRP Exhibits antiplatelet activity	Potentially decreases plasma concentration of all DOACs Potentially increases the risk of bleeding when used concomitantly with DOACs
Salvianolic acid *	*M. spicata* (*Spearmint*) *S. miltiorrhiza* (*Danshen*)	Inhibition of cytochrome P450 (1A2, 3A4) and P-gp, induction of BCRP	Potentially increases plasma concentration of dabigatran, edoxaban and betrixaban
Schisandrin B *	*S. chinensis* (*Magnolia vine*)	Inhibition of cytochrome P450 (3A4, 3A5) and P-gp	Potentially increases plasma concentration of all DOACs
Silymarin *	*Silybum marianum* (*Asteraceae*)	Inhibition of CYP3A4 and P-gp	Potentially increases plasma concentration of all DOACs
β-Sitosterol *	*A. lancea* *C. pluricaulis* (*Shankhpushpi*) *M. peregrina* (*Ben tree*) *M. pruriens* (*Velvet bean*)	Inhibition of BCRP	Potentially increases plasma concentration of apixaban and rivaroxaban
Stigmasterol *	*A. lancea*	Inhibition of cytochrome P450 (3A4, 3A5) and P-gp	Potentially increases plasma concentration of all DOACs
Tannic acid *	*T. chebula* (*Chebulic myrobalan*)	Inhibition of cytochrome P450 (1A2, 3A4, 2B6) and P-gp	Potentially increases plasma concentration of all DOACs
Tanshinone I *	*S. miltiorrhiza* (*Danshen*)	Inhibition of P-gp and BCRP	Potentially increases plasma concentration of all DOACs
Tanshinone IIA *	*S. miltiorrhiza* (*Danshen*)	Inhibition of P-gp and BCRP	Potentially increases plasma concentration of all DOACs
Tenacissimoside A	*Marsdeniatenacissima* (*Asclepiadaceae*)	Inhibition of P-gp	Potentially increases plasma concentration of all DOACs
Tetrandrine *	*Stephania tetrandra* (*Menispermaceae*)	Inhibition of CYP3A4 and P-gp	Potentially increases plasma concentration of all DOACs
Thymol, γ-terpinene	*C. copticum* (*CarumAjowan*)	Inhibition of CYP3A4	Potentially increases plasma concentration of rivaroxaban, apixaban and edoxaban
Timosaponin AIII *	*A. asphodeloides* (*RhizomaAnemarrhenae*)	Inhibition of P-gp	Potentially increases plasma concentration of all DOACs
Trigonelline *	*T. foenum-graecum* (*Fenugreek*)	Induction of BCRP	Potentially decreases plasma concentration of apixaban and rivaroxaban
Ursolic acid *	*R. officinalis* (*Rosemary*)	Inhibition of cytochrome P450 (1A2, 3A4, 2C8, 2C9, 2C19, 2D6) and BCRP	Potentially increases plasma concentration of apixaban, rivaroxaban and edoxaban
Valerenic acid *	*V. officinalis* (*Valerian*)	Inhibition of CYP3A4	Potentially increases plasma concentration of rivaroxaban, apixaban and edoxaban
Vauqueline	*A. sinensis* (*Dong quai*)	Inhibition of P-gp	Potentially increases plasma concentration of all DOACs
Vitamin E		Exhibits antiplatelet activity	Potentially increases the risk of bleeding when used concomitantly with DOACs
	*F. multiflora* (*Fo-ti-root*)	Inhibition of cytochrome P450 (1A2, 2C9, 2C19, 3A4)	Potentially increases plasma concentration of rivaroxaban, apixaban and edoxaban
	*Lamiaceae* (*Scutellaria*)	Inhibition of CYP3A4 and induction of P-gp	Potentially decreases plasma concentration of dabigatran and betrixaban
	*Sucralose*	Induction of CYP3A4 and P-gp	Potentially decreases plasma concentration of all DOACs
	*U. tomentosa* (*Cat’s claw*)	Inhibition of CYP3A4	Potentially increases plasma concentration of rivaroxaban, apixaban and edoxaban

* Phytochemicals found in food supplements. Abbreviations: BCRP, breast cancer resistance protein; MATE1, multidrug and toxin extrusion protein 1; MATE2K, multidrug and toxin extrusion protein 2K; P-gp, P-glycoprotein; CYP3A4, cytochrome P450 3A4.
